# MicroRNA Expression for Early Prediction of Late Occurring Hematologic Acute Radiation Syndrome in Baboons

**DOI:** 10.1371/journal.pone.0165307

**Published:** 2016-11-15

**Authors:** Matthias Port, Francis Herodin, Marco Valente, Michel Drouet, Reinhard Ullmann, Sven Doucha-Senf, Andreas Lamkowski, Matthäus Majewski, Michael Abend

**Affiliations:** 1 Bundeswehr Institute of Radiobiology, Munich, Germany; 2 Institut de Recherche Biomedicale des Armees, Bretigny-sur-Orge, France; Georgetown University, UNITED STATES

## Abstract

For effective medical management of radiation-exposed persons after a radiological/nuclear event, blood-based screening measures in the first few days that could predict hematologic acute radiation syndrome (HARS) are needed. For HARS severity prediction, we used microRNA (miRNA) expression changes measured on days one and two after irradiation in a baboon model. Eighteen baboons underwent different patterns of partial or total body irradiation, corresponding to an equivalent dose of 2.5 or 5 Gy. According to changes in blood cell counts (BCC) the surviving baboons (n = 17) exhibited mild (H1-2, n = 4) or more severe (H2-3, n = 13) HARS. In a two Stage study design we screened 667 miRNAs using a quantitative real-time polymerase chain reaction (qRT-PCR) platform. In Stage II we validated candidates where miRNAs had to show a similar regulation (up- or down-regulated) and a significant 2-fold miRNA expression difference over H0. Seventy-two candidate miRNAs (42 for H1-2 and 30 for H2-3) were forwarded for validation. Forty-two of the H1-2 miRNA candidates from the screening phase entered the validation step and 20 of them showed a statistically significant 2–4 fold up-regulation relative to the unexposed reference (H0). Fifteen of the 30 H2-3 miRNAs were validated in Stage II. All miRNAs appeared 2–3 fold down-regulated over H0 and allowed an almost complete separation of HARS categories; the strongest candidate, *miR-342-3p*, showed a sustained and 10-fold down-regulation on both days 1 and 2. In summary, our data support the medical decision making of the HARS even within the first two days after exposure where diagnostic tools for early medical decision are required but so far missing. The miRNA species identified and in particular *miR-342-3p add to the previously identified mRNAs and complete the portfolio of identified mRNA and miRNA transcripts for HARS prediction* and medical management.

## Introduction

In a large-scale radiological emergency, early detection of exposed individuals would be required in order to evaluate the extent of radiation injuries and, as necessary, provide appropriate treatment [1]. Estimates of the absorbed dose aid in determining risk for later occurring acute (up to 60 days after irradiation) or chronic (months and years after irradiation) health effects. In particular, after high-dose exposure (≥ 2 Gy single whole body dose), severe acute health effects (acute radiation syndrome, ARS) will occur and early diagnosis within 1–3 days after exposure is essential to hospitalize exposed individuals in specialized clinics and to quickly initiate appropriate treatment. For instance, treatment with cytokines (Granulocyte-Colony Stimulating Factor (G-CSF)) should start within the first day after exposure and appropriate diagnostic tools are required and missing ([2],[3]). It is important to identify the few highly exposed individuals from among many who believe they were exposed (the worried well), to avoid depletion of clinical resources.

In the absence of physical dosimetry (e.g. improvised nuclear device), biological changes after radiation exposure are used for individual dose estimates. For instance, scoring dicentric chromosomes represents the gold standard in biological dosimetry [4]. Physical and biological dosimetry that estimate *exposure dose* are thought as an appropriate way to extrapolate the *effect* caused by the exposure. The higher the dose, the stronger will be the health effects, including ARS severity. However, the estimate of exposure dose becomes difficult when dealing with heterogeneous instead of homogeneous radiation exposures. For example, in the case of partial vs. total body radiation exposure, or when dealing with different dose rates or different radiation exposure qualities (gamma, alpha, neutron), or combined internal (incorporation of radionuclide) or external radiation exposure. In principle, a calibration curve for each of these aspects alone or combined would be required for biological dosimetry. Dose estimates based on physical dosimetry rely on these modifiers of exposure. Such estimates of an equivalent whole body dose require the agreement of experts, as in many radiation exposures in the past e.g. atomic bombs in Hiroshima and Nagasaki in 1945 [5] or radiation accidents such as Goiania in 1987 [6,7], Chernobyl in 1986 [8–10] or Fukushima 2011 [11–13]. Experience has shown that for these large groups, dose estimates may be modified later due to updated knowledge. For instance, at Hiroshima/Nagasaki the neutron exposure was higher than initially thought, and was part of the impetus to revise the physical dosimetry (DS02; dosimetry system established in 2002) years after the exposure (reviewed in [5]). Also, there is doubt about the using of exposure to base such extrapolations of the effect. As a result, in approaches like METREPOL (MEdical TREatment ProtocOLs), early clinical signs and symptoms are used for prediction of the weeks later occurring haematologic ARS (HARS) [14]. METREPOL categorizes HARS into five severity degrees based on blood cell count (BCC) changes in the weeks that follow the exposure: no HARS (H0°), low (H1°), medium (H2°), severe (H3°) and fatal (H4°). With the decrease in granulocytes and thrombocytes in the peripheral blood, the haematological syndrome of ARS is characterized mainly by immune suppression and haemorrhage over time.

We hypothesized the depletion of BCC would be preceded by changes in miRNA expression and, therefore, could serve as an indication of the weeks later occurring HARS severity score. In collaboration with the French Army Biomedical Research Institute, we assessed blood samples obtained from irradiated baboons taken before (day 0, and serving as H0°) and 1 and 2 days after partial/total body exposure. BCC were measured in these baboons 7–203 days after exposure, allowing for estimation of future HARS severity.

In previous work [15] we used blood samples taken before and 1–2 days after exposure and performed a whole genome screening on protein coding genes (messenger RNA (mRNAs)). We identified genes associated with late occurring HARS severity score. These genes were then validated using qRT-PCR. We found 29 candidate genes that were useful to predict H0°, H1-3° or H2-3° HARS. Encouraged by these results, we considered that the prediction might be improved by examining changes on the post-transcriptional level for miRNAs that control mRNA gene expression.

In the current study, we examined 667 miRNAs in a similar two Stage study design as we did for the protein coding mRNAs. Herein we do not focus on the *dose-to- expression* relationship, but rather the *gene-to-disease relationship*. We report our findings to predict the effect (disease) based on early changes in miRNA expression after irradiation.

## Materials and Methods

### Animals

The baboon model was chosen as a highly relevant model based on its genetic and physiological proximity to humans and its size, which allows to take into account the impact of corpulence in dose distribution. Eighteen baboons were bred by the Centre National de la Recherche Scientifique (Rousset sur Arc, France) for the purpose of biomedical research. The male baboons had an average age of 8.1 years (+/- 3.3 years) and weighed 23.7 ± 5.2 kg. In the nonhuman primate facility of the French Army Biomedical Research Institute, the baboons were placed in individual cages (1.5m x 1.5m x 2m, height) at 21°C (+/- 1°C) and 55% humidity (+/- 5%). The animals received fresh fruit (Au Jardin De Provence, Grenoble, France) and solid food twice a day (Old World Monkey chunks, Special Diet Service, Argenteuil, France), and had access to water *ad libitum*. During the period of radiation-induced transient anorexia (taking place within the first 15 days after irradiation in three baboons) animals received a completely balanced diet with appetizing fruit and fruit juices.

The environmental enrichment provided to the baboons relied on twice daily interactions with animal keepers (speaking to and playing with baboons for 2x30 min) in addition to the daily clinical examination performed by the principal investigator. Four baboons in individual cages were placed in each room for ensuring sufficient social activity. Appropriate toys were available in the cages. A full-time attending veterinarian (AV) was employed in the non-human primate facility. A written program of veterinary care was applied with regularly scheduled visits. The AV monitored the baboons for a humane end point. Specifically, the veterinary surgeon in charge of animal welfare fulfilled a mission of council and inspection to ensure that nonhuman primates were provided supportive care in ways that minimize fear, pain, stress, and suffering. Accordingly, two baboons showing signs of pain received buprenorphine (Vetergesic Multidose, Sogeval, Sheriff Hutton, UK). Furthermore, animals received fresh, whole blood transfusion after irradiation when e.g. platelets lowered below 20 x 10^9^/L or after observation of petechiae. Ampicillin (50 mg/kg/day) and gentamycin (1.5 mg/kg/day) were provided during neutropenia (ANC lower than 0.5 x 10^9^/L) and cefalexin (1 mg/kg/day for 3 days) during prolonged febrile periods above 39°C. All surviving baboons were sacrificed at day 200 after irradiation using intravenous injection of Natrium Pentobarbital (100 mg/kg).The experiment was approved by the French Army Animal Ethics Committee (No 2010/12.0). All baboons were treated in compliance with the European legislation related to animal care and protection in order to minimize pain and stress. One baboon irradiated with a sub lethal dose unexpectedly died from pancytopenia on day 24 after radiation in spite of treatment (2 blood transfusions). He was also given treatment against pain. As the absorbed radiation dose was not 100% lethal it emphasizes the impact of individual variance in radio sensitivity.

### Irradiation

The animals were anesthetized with a combination of tiletamine and zolazepam (6 mg/kg intramuscularly, Zoletil 100; Virbac, Carros, France) before irradiation. Then, the baboons were placed in restraint chairs, sitting orthogonally, front to a horizontal and homogeneous field of gamma rays delivered by a ^60^Co source (IRDI 4000; Alsthom, Levallois, France) to perform either total body irradiation (TBI) or partial body irradiation (PBI). Time in restraint chairs was acceptable under conditions of the experiment and animal behaviour was monitored during irradiation using a video camera. In order to attain different patterns of PBI, a 20 cm thick lead screen was used to shield different parts of the body as detailed in [16]. Two baboons were exposed to 5 Gy TBI and two others to 2.5 Gy TBI. Eight different exposure patterns were simulated and two baboons were exposed per pattern which summed up to 16 baboons receiving PBI (for details see [16]) corresponding to an equivalent TBI dose of 2.5 or 5 Gy. Two dose rates were used (8 cGy/min for 5 Gy TBI and 5 Gy 50% PBI, and 32 cGy/min for all other situations) because the Cobalt 60 source was changed during this study. To achieve the same homogeneous radiation field whatever the dose rate, all baboons were irradiated at the same distance from the source. Consequently, radiation exposures lasted between 8 min and 62 min. The mid-line tissue (right anterior iliac crest) dose in air was measured with an ionization chamber. Delivered doses were controlled by alumina powder thermoluminescent dosimeters placed on different cutaneous areas (thorax, thoracic and lumbar vertebrae, head, tibia, femur, femoral head, for details see [16]). Since animals were exposed using either partial or total body irradiation (for details see [15] this resulted in whole body equivalent doses of 0 Gy for H0° HARS (blood samples taken before irradiation) and 2.5–5 Gy for baboons developing a mild H1-2° HARS disease and the same dose range of 2.5–5.0 Gy was observed for baboons developing a more aggravated H2-3° HARS disease (Table 1).

**Table 1:** Overview on radiation exposure and blood samples used for screening and validation purposes.

	whole body equivalent dose (Gy)	screening			validation		
		days after irr.			days after irr.		
HARS°		0	1	2	0	1	2
0°	0	5			11		
1–2°	2.5–5.0		4	4		0	0
2–3°	2.5–5.0		5	5		7	8

### Blood collection, determination of HARS severity scores and other clinical symptoms

Using changes in BCC, the severity scores (0°, unexposed; 1–4°, low to severe degree) of HARS was determined using the criteria of METREPOL [14]. HARS scoring was based on changes in differential blood counts taken at up to 13 time points over the course of 7–203 d after exposure [15]. A time course such as that was required to perform the HARS scoring based on the METREPOL approach [14]. Often changes in lymphocyte counts or thrombocytes over time indicated HARS differing from each other so that intermediates between, e.g. HARS 2–3° had to be defined. In contrast, whole blood samples for miRNA gene expression measurements were taken only before irradiation (0 h) and at 1 day and 2 days after irradiation. These samples were evaluated for radiation-related changes in miRNA expression to predict HARS score over the ensuing 7 to 203 days. In total there were 49 samples obtained on days zero and days one and two with 23 samples used for Stage I (screen) and the remaining samples (n = 26) for the validation in Stage II (Table 1). Six baboons experienced further clinical symptoms including transient (2 h) moderate vomiting (4 baboons), erythema and hair loss (n = 3), and a transient weight loss of 4–10% (3 baboons).

Animals were exposed using either partial or total body irradiation (for details see [15]). This resulted in whole body equivalent doses of 0 Gy for H0° HARS (blood samples taken before irradiation) and 2.5 Gy for baboons developing a mild H1-2° HARS disease and 2.5–5.0 Gy for baboons developing a more aggravated H2-3° HARS disease. The following columns depict the number of blood samples available for H0° (day 0), H1-2° and H2-3° HARS categories during the first two days after exposure and used for screening (Stage I) and validation (Stage II) according to the split two Stage study design. There were 23 blood samples used for Stage I (screening) and 26 samples for Stage II (validation). With 17 animals examined at three time points altogether 51 blood samples would be expected, but sufficient amounts of RNA were left for 49 samples from previous examinations.

### RNA extraction and quality control

Whole blood samples (2.5 ml) were processed following the PAXgene Blood RNA system (BD Diagnostics, PreAnalytiX GmbH, Hombrechtikon, Switzerland). In brief, blood was drawn into a PAXgene Blood RNA tube at the French Army Biomedical Research Institute. The tube was gently inverted (10 times), stored at room temperature overnight then at -20°C. After all samples were collected, the PaxGene tubes were sent to Germany for further processing. After thawing, washing and centrifugation, cells in the supernatant were lysed (Proteinase K) followed by addition of Lysis/Binding Solution taken from the mirVana Kit (Life Technologies, Darmstadt, Germany). With the mirVana kit total RNA, including small RNA species, was isolated by combining a Phenol-Chloroform RNA precipitation with further processing using a silica membrane. After several washing procedures DNA residuals became digested on the membrane (RNAse free DNAse Set, Qiagen, Hilden, Germany). RNA was eluted in a collection tube and frozen at -20°C. Quality and quantity of isolated total RNA were measured spectrophotometrically (NanoDrop, PeqLab Biotechnology, Erlangen, Germany). RNA integrity was assessed by the 2100 Agilent Bioanalyzer (Life Science Group, Penzberg, Germany) and DNA contamination was controlled by conventional PCR using an actin primer. We used only RNA specimens with a ratio of A_260_/A_280_ ≥ 2.0 (Nanodrop) and RNA integrity number (RIN) ≥ 7.5 for whole genome microarray (IMGM Laboratories, Martinsried, Germany) or RIN ≥ 7.3 for qRT-PCR analyses.

### Stage I screening: qRT-PCR, 384-well format low density array (LDA)

The *screening* for differentially expressed miRNAs was performed on 23 RNA samples with a subsequent range of HARS scores (H0° n = 5, H1-2° n = 2x4, on days 1 and 2 after exposure, H2-3° n = 2x5, on days 1 and 2 after exposure, Table 1).

A commercially available 384-well qRT-PCR platform (LDA) was used that provided the simultaneous detection of 380 different miRNAs. Two different LDAs (type A and B) were combined so that the detection of 667 miRNA species was possible. Aliquots from each RNA sample (in general 2 μg total RNA/LDA type A/B) were reversely transcribed without preamplification over three hours using *“Megaplex pools without preamplification l for microRNA expression analysis protocol”*. Using different sets of primers, two kinds of cDNAs suitable for each of both LDAs were created. In a second step, the whole template cDNA and 450 μl 2x RT-PCR master mix were adjusted to a total volume of 900 μl by adding nuclease free water, and aliquots of 100 μl were pipetted into each fill port of a 384-well human LDA. Cards were centrifuged twice (12,000 rpm, 1 min, Multifuge3S-R, Heraeus, Germany), sealed, transferred into the 7900 RTQ-PCR instrument and a specific RTQ-PCR protocol was run over two hours using the 384-well LDA format.

We used a previously established upper limit of the linear-dynamic range of our qRT-PCR, threshold cycles (CT) ≤ 30 [17]. Normalization was performed using the median miRNA expression on each LDA separately, because this proved to be the more robust and slightly more precise method compared to a normalization approach using a housekeeping miRNA species provided on the LDA (data not shown). The median miRNA expression was subtracted from the CT-value of each of the spotted genes, following the ΔCT−quantitative approach for normalization purposes. Only two-fold ratios (≥2/≤0.5) were considered to represent differentially expressed genes.

All technical procedures for qRT-PCR were performed in accordance with standard operating procedures implemented in our laboratory in 2008 when the Bundeswehr Institute of Radiobiology became certified according to DIN EN ISO 9001/2008. All chemicals for qRT-PCR using TaqMan chemistry were provided by Life Technologies, Darmstadt, Germany.

Candidate miRNAs had to be expressed in ≥ 75% of the examined blood samples per HARS group, normalized miRNA expression had to differ ≥ 2-fold over H0° and either the mean miRNA expression among HARS groups had to be statistically significant (t-test, p<0.05) or the concordance (explained in the statistic part) to discriminate HARS groups had to be ≥ 75%.

Due to the explorative nature of this study and the small sample size we did not correct for multiple comparisons on the screening step (Stage I) of the study. We did multiple comparison adjustment (Bonferroni correction) in the validation step (Stage II) of our study.

### Stage II: Validation of Stage I candidate genes via qRT-PCR (LDA)

For validation of our miRNAs from the Stage I screen, we used the same miRNA LDA (type A&B) as in Stage I. Among the 667 miRNAs evaluated, 72 candidates were identified in Stage I; 42 miRNAs appeared associated with 1–2 degree HARS. Since no 1–2 degree HARS samples remained, we could not validate these in Stage II. There were 30 miRNAs associated with 2–3 degree HARS that remained, so we used them for validation in Stage II.

During the validation stage we selected for miRNAs showing similar regulation (up-regulation or down-regulation) as in Stage I (screening). We also required that the miRNA expression was statistically significant (p<0.05), showed a ≥ 2-fold difference in miRNA gene expression over H0°, and had a concordance (explained in the statistic part) ≥ 75%.

### Statistical analysis, Stage I and II

Using the normalized miRNA expression results from Stages I and II, we examined minor-mild (H1-2°) vs none (H0°) HARS, and severe (H2-3°) vs none. Descriptive statistics (n, mean, standard deviation, min, max) and p-values (t-test) were calculated for each of the variables (candidate miRNAs) and per time point (days 0, 1, or 2). Unconditional logistic regression was used to analyze each miRNA separately (univariate) by considering HARS (grouped into grades 1–2° and 2–3°) as binary outcome variables with H0° serving as the reference. Odds ratios (OR), 95% confidence intervals (95% CI) and corresponding p-values (Wald Chi-Square) as well as the concordance were calculated. Concordance assesses the ability of the predictive model to distinguish between healthy (H0°) animals and baboons’ subsequently showing degrees of clinical HARS. A concordance of 75% indicates that 75% of the samples could be correctly assigned to one of the two binary categories (H0° or the higher HARS severity group) using the predictive model. In the case of complete separation of data sets (concordance equals 100%), missing p-values using logistic regression analysis were replaced by p-values (Chi-Square) generated by frequency distribution comparisons. All calculations were performed using SAS (release 9.2, Cary NC, USA).

### Bioinformatic approach: Analysis of miRNA-mRNA interactions

We investigated whether there were potential regulatory interactions between the 33 HARS specific non-coding miRNAs identified in this study and the 29 coding genes (mRNAs) with HARS associated regulation, which we defined in our previous study [15]. For this purpose we have downloaded a dataset encompassing 22.9 million predicted miRNA-mRNA interactions from the TargetScan 7.0 database (http://www.targetscan.org/; [18]) and filtered it based on our lists of HARS related mRNA (n = 29) and miRNAs (n = 33). From the remaining miRNA-mRNA interactions, we selected those that were predicted based on the human genome sequence as qPCR and microarray analysis in our study do also refer to the human genome. Results of this screen, and corresponding annotation data such as RNA expression levels and cumulative weighted context++ scores, which indicate how effectively a given miRNA may target mRNAs [19], were visualized in Cytoscape [20].

## Results

### Defining hematologic response categories

Based on changes of the lymphocyte, granulocyte and thrombocyte counts examined 7–203 days after exposure, HARS scores were determined according to the criteria of METREPOL (for details see [15]). We found METREPOL categories for HARS were difficult to apply, since e.g. granulocyte counts during follow-up could reflect H2°, while thrombocytes appeared more representative of a H1 degree HARS. As a result we ultimately classified 17 H0°, four H1-2° and 13 H2-3 degree HARS [15].

### Material available for the two Stage study design

Due to unexpected sudden death after irradiation, one out of the 18 baboons had to be excluded, leaving 17 baboons eligible for analysis.

During the *screening approach at Stage I*, we used 23 blood samples (Table 1). Blood samples collected before irradiation from five baboons were selected randomly and represented H0 degree HARS. Four baboons developed H1-2°, so these four blood samples were selected from days 1 and 2 after irradiation for screening purposes (n = 8). Ten out of 35 blood samples were selected randomly from another five baboons which developed H2-3° from days 1 and 2.

For the *independent methodological validation at Stage II*, we used only available blood samples not used for the screening approach (n = 26, Table 1). The number of samples was 11 for H0° and 15 for H2-3° (7 samples 1 day and 8 samples 2 days after irradiation), respectively.

### Stage I: RNA isolation and screening results using qRT-PCR measurements

From 2.5 ml whole blood we isolated 10, 8.5 and 6.3 μg total RNA on average before irradiation and 1 and 2 days after irradiation, respectively. RNA integrity (RIN) with a mean value of 8.6 (stdev +/- 0.6, min 7.3, max 9.5) suggested high quality RNA sufficient for running qRT-PCR.

**Fig 1:**
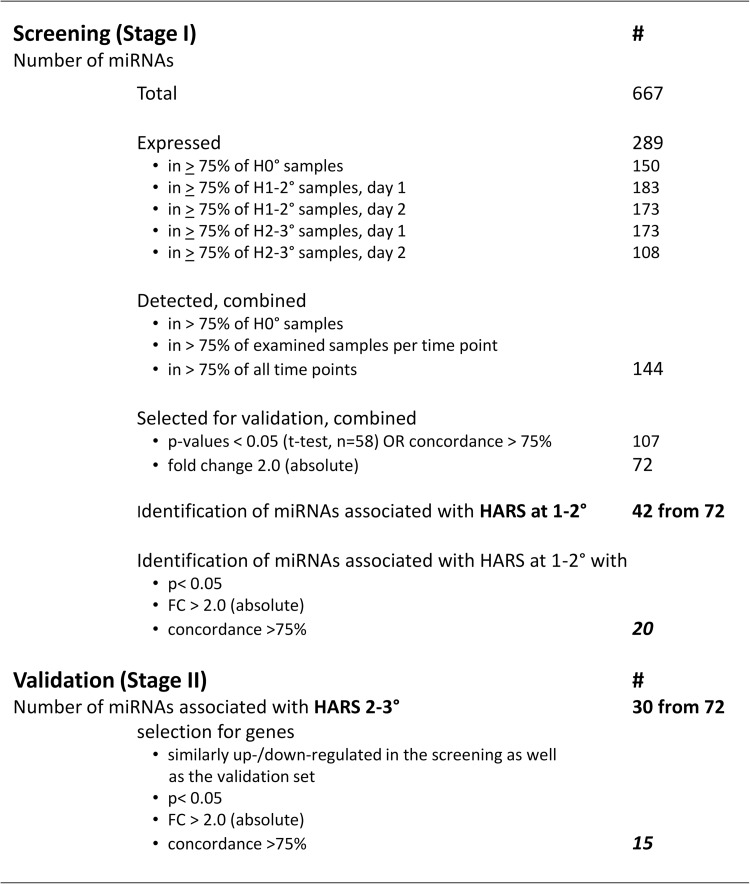
Screening and validation for samples eligible in Stages I and II of the two-Stage split study design. Shown are the numbers of miRNAs included at each step of the eligibility process. Abbreviation: FC, fold-change. miRNA gene expression values within the linear-dynamic range of the methodology are called expressed. Preselected candidate miRNAs (detected miRNAs) had to fulfill certain criteria which became more defined for the selected candidates forwarded to Stage II.

**Fig 2:**
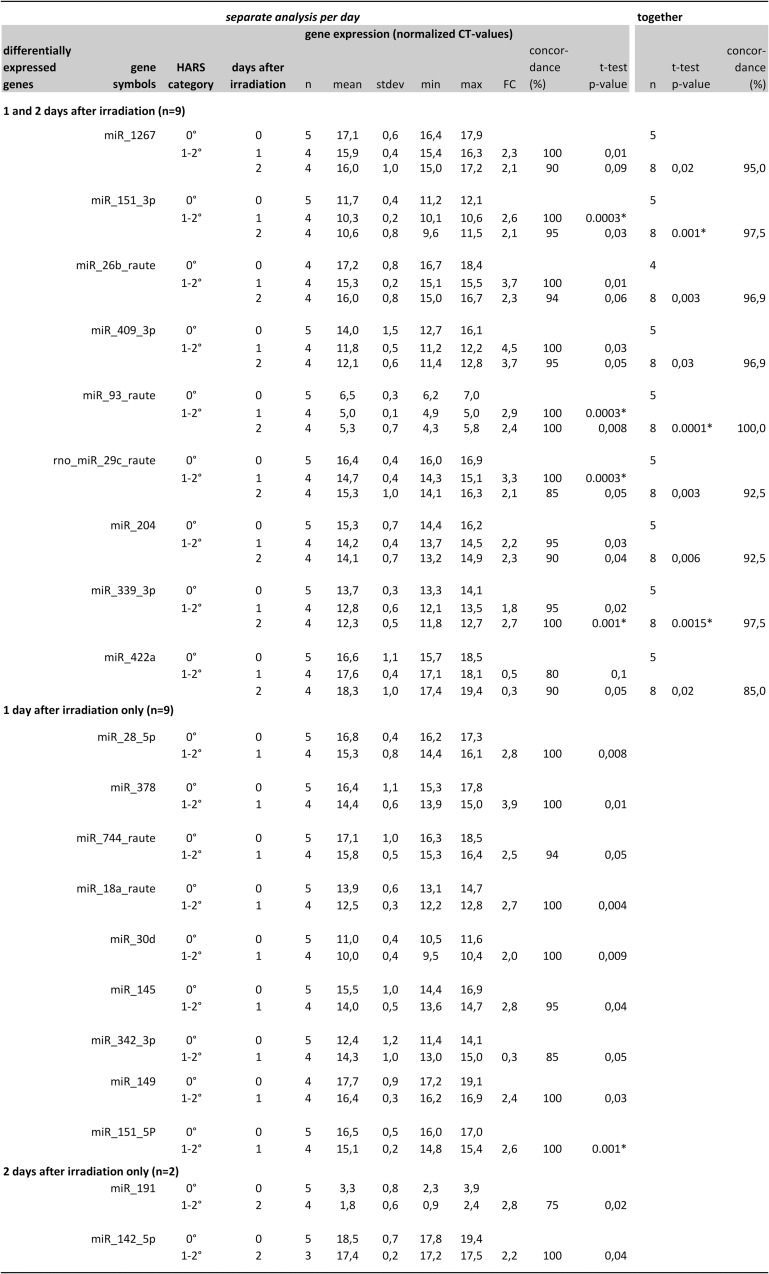
The Figure summarizes qRT-PCR results (normalized threshold cycle [CT]-values) from Stage I (screening) comparing H1-2° severity of the hematological acute radiation syndrome (HARS) with H0° (unexposed, reference) at days 1 and 2 after radiation exposure. Descriptive statistics reflect the distribution and the fold-change (FC) difference among miRNAs differentially expressed at H1-2°, with H0° as the reference. Mean values of both HARS groups were compared with the Students t-test. Concordance was calculated using logistic regression analysis. In the case of complete separation of data sets (concordance equals 100%), missing p-values using logistic regression analysis were replaced by p-values (Chi-Square) generated by frequency distribution comparisons. Samples showing the same direction in the FC on both days were merged into one category and analyzed together (last part of the figure). Genes with p-values <0.0025 survived Bonferroni correction for multiple comparisons (p = 0.05/20 miRNAs/hypothesis ~ 0.0025) and are marked with an asterisk.

From 667 miRNAs measured in Stage I, 289 miRNA were detectable/expressed in all 23 samples examined (Fig 1). The number of miRNAs expressed in ≥ 75% at 1 and 2 days after irradiation and in ≥ 75% of all three HARS groups (H0°, H1-2° and H2-3°) combined was reduced to 144 miRNAs. Based on a p≤0.05 (t-test) or a concordance ≥ 75% and an absolute fold-difference ≥ 2.0 (relative to H0°), 72 miRNAs (42 for H1-2° and 30 for H2-3°) were eligible for Stage II validation. However, H1-2° HARS could not be validated in Stage II since all 4 samples were already used in Stage I. In Stage I we found 20 of 42 miRNAs were statistically significant, had 2–4 fold mostly up-regulated miRNAs, and a concordance between 90–100% for discriminating from H0° (Fig 2). Nine of these 20 miRNAs showed a sustained expression over both days after exposure. The other 11 miRNAs appeared differentially expressed on either the first (n = 9) or the second day (n = 2) after exposure. Five out of 20 miRNA survived Bonferroni correction for multiple comparisons.

**Fig 3:**
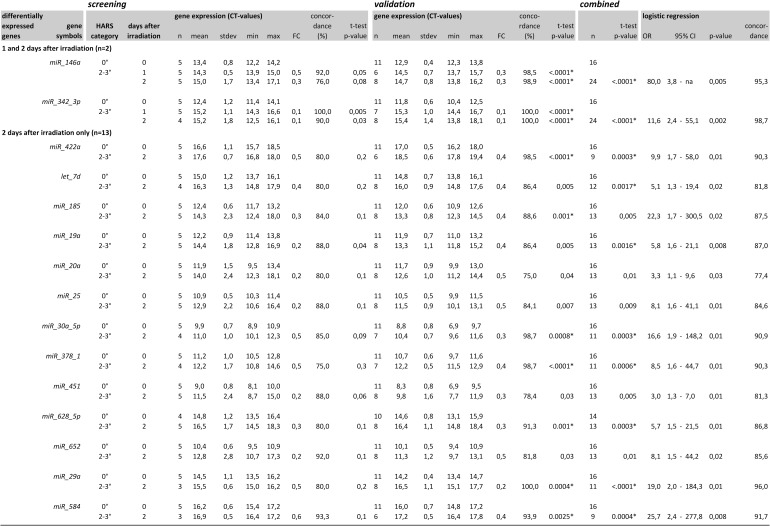
Summarized qRT-PCR results (normalized threshold cycle [CT]-values) from Stage I (screening), Stage II (validation) and a combined analysis on all samples (Stage I and Stage II samples together). MiRNAs values measured before irradiation (H0°) were compared with corresponding miRNA values measured at days 1 and 2 after irradiation in baboons developing a more aggravated H2-3° HARS. Descriptive statistics reflect the distribution and the fold-change (FC) difference among miRNAs differentially expressed at H2-3° with H0° as the reference. Mean values of both HARS groups were compared employing the Students t-test. Concordance was calculated using logistic regression analysis. In the case of complete separation of data sets (concordance equals 100%), missing p-values using logistic regression analysis were replaced by p-values (Chi-Square) generated by frequency distribution comparisons. Samples showing the same direction in the FC on both days were merged into one category and analyzed combined (last part of the figure). Genes with p-values <0.0033 survived Bonferroni correction for multiple comparisons (p = 0.05/15 miRNAs/hypothesis ~ 0.0033) and are marked with an asterisk.

For H2-3° prediction, 30 miRNAs were identified in Stage I as eligible for Stage II (data not shown). In Stage II, half of them showed similar characteristics as the samples used for the independent validation and are presented in Fig 3. All 15 miRNAs from the Stage I screen showed a 2–3 fold down-regulation over H0°, but *miR-342-3p* had a sustained and 10-fold down-regulation at 1 and 2 days after exposure. The second miRNA that was 2–3 fold down-regulated on both days after exposure was *mir-146a* (Fig 3). The other 13 miRNAs were differentially expressed on the second day only. The concordance ranged between 80–95% for most of the miRNAs.

### Stage II: validation using qRT-PCR measurements

**Fig 4:**
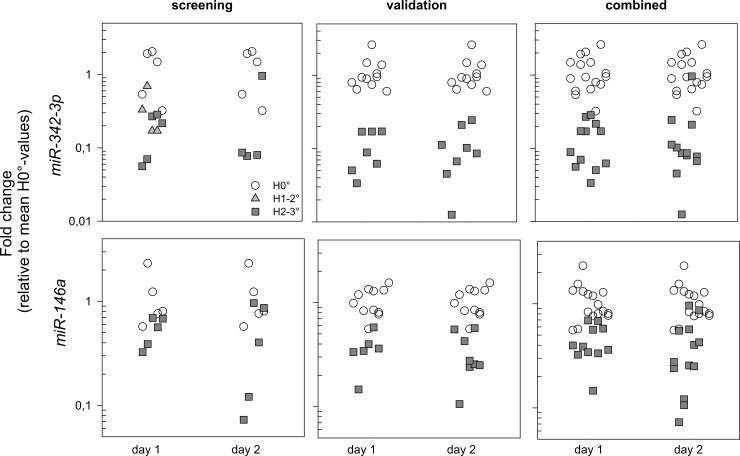
Plots of fold changes (FC) for *miR-342-3p* and *miR-146a* relative to the mean H0°-values are shown separately for each blood sample taken on day 1 and day 2 after exposure. The plots correspond with data shown in Fig 2 for these miRNAs. FCs generated on the samples for the screening (left side), the independent validation (middle) and all samples combined (right side) are presented. A jitter plot was chosen to avoid overlap of similar data. Circles, triangles and squares represent H0°, H1-2° and H2-3° HARS, respectively.

For H2-3° prediction, 15 out of 30 miRNAs were validated in Stage II. All miRNAs appeared 2–3 fold down-regulated over H0° and each were able to distinguish between the HARS categories. Again, *miR-342-3p* showed a sustained, significant (p<0.0001) and 10-fold down-regulation at days 1 and 2 after exposure in Stage II and combined over both Stages as well (Figs 3 and 4). A sustained, significant (p<0.0001) and 3-fold down-regulation over H0° was observed for *miR-146a* at both days in the validation set and combined over both Stages (Figs 3 and 4). With the increased sample size in Stage II, p-values decreased to values < 0.0033 (0.05/15) so that 9 out of 15 miRNAs survived Bonferroni correction for multiple comparisons. When combining samples used for the screen and the validation, 10 miRNAs survived the Bonferroni correction and the concordance for most miRNAs appeared close to or above 90% (Fig 3).

Of note, significant gene-to-disease associations as shown in Figs 2 and 3 at the same time do reflect significant dose-to-gene associations, since H0° represents unexposed and H1-2° or H2-3° HARS are representative of exposed samples.

### Candidate miRNA over time and HARS severity

Based on the screening for prediction of HARS 1–2°, nine (day 1), two (day 2) and nine miRNAs (two days combined) could be identified (Table 2). Almost all miRNAs were 2–4 fold up-regulated over H0°. Two miRNAs, *miR-342-3p* and *miR-422a*, were down-regulated for both HARS 1–2° and HARS 2–3° suggesting they are predictive for any HARS (H1-3°). In particular, *miR-342-3p* decreased 3-fold compared to H0° before HARS 1–2° developed and 10-fold before HARS 2–3° developed. The miRNA *miR-342-3p* appeared to be the best candidate for prediction of HARS 2–3°. There were no miRNAs that could predict HARS 2–3° on day 1 only, but 13 miRNAs predicted HARS 2–3° on day 2 and two (*miR-342-3p* and *miR-146a*) on both days after exposure (Table 2).

**Table 2:** Summary of candidate miRNAs predicting different degrees of HARS.

HARS 1–2°						HARS 2–3°					
1st day (n = 9)		2nd day (n = 2)		1st & 2nd day (n = 9)		1st day (n = 0)		2nd day (n = 13)		1st & 2nd day (n = 2)	
gene symbol	fold change	gene symbol	fold change	gene symbol	fold change	gene symbol	fold change	gene symbol	fold change	gene symbol	fold change
*miR_145*	2.8	*miR_142_5p*	2,2	*miR_1267*	2.3 & 2.1			*let_7d*	0.4	*miR_146a*	0.3 & 0.3
*miR_149*	2.4	*miR_191*	2,8	*miR_151_3p*	2.6 & 2.1			*miR_185*	0.4	***miR_342_3p***	**0.1 & 0.1**
*miR_151_5P*	2.6			*miR_204*	2.2 & 2.3			*miR_19a*	0.4		
*miR_18a_raute*	2.7			*miR_26b_raute*	3.7 & 2.3			*miR_20a*	0.5		
*miR_28_5p*	2.8			*miR_339_3p*	1.8 & 2.7			*miR_25*	0.5		
*miR_30d*	2			*miR_409_3p*	4.5 & 3.7			*miR_29a*	0.2		
***miR_342_3p***	**0.3**			***miR_422a***	**0.5 & 0.3**			*miR_30a_5p*	0.3		
*miR_378*	3.9			*miR_93_raute*	2.9 & 2.4			*miR_378_1*	0.4		
*miR_744_raute*	2.5			*rno_miR_29c_raute*	3.3 & 2.1			***miR_422a***	**0.4**		
								*miR_451*	0.3		
								*miR_584*	0.4		
								*miR_628_5p*	0.3		
								*miR_652*	0.5		

Summary of 33 candidate miRNAs that predict H1-2° HARS (lower side) and H2-3° HARS relative to H0° at days 1 or 2 or both days after radiation exposure. Bold miRNA names represent two differentially expressed miRNAs at H1-2° and H2-3° (H1-3°). All other miRNAs are mutually exclusively differentially expressed either at H1-2° or H2-3°.

### Analysis of miRNA-mRNA interactions

**Fig 5:**
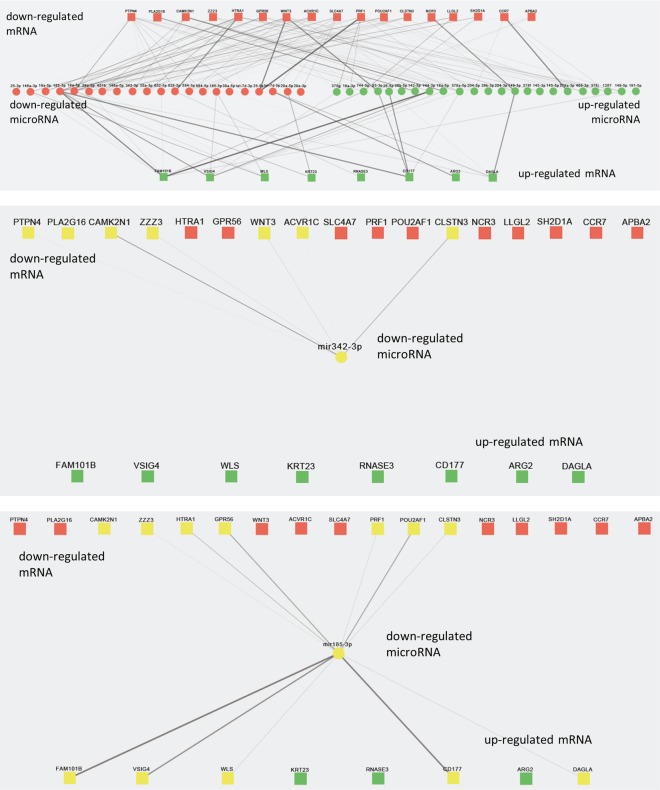
Graphical summary of predicted miRNA-mRNA interactions. The miRNAs and mRNAs, represented by circles and squares, respectively, are grouped according to their expression levels with down-regulated mRNAs depicted in the top row, miRNAs in the middle row and down-regulated genes at the bottom. Visibility (thickness) of the lines connecting miRNAs and mRNAs is defined by the corresponding cumulative weighted context++ score. Context++ scores indicate how effective the miRNA might regulate the targeted mRNA. Visualization was done by means of Cytoscape. The upper panel depicts all 211 interactions, 29 mRNA (from a previous study) and 33 miRNAs from the present study. Predicted interactions of the most promising *miR342-3p* miRNA are depicted in the middle panel and expected reciprocal and stronger miRNA (*miR185-3p*)–mRNA interactions are shown in the lower panel.

Fig 5 (upper panel) depicts all 211 miRNA-mRNA interactions (lines) that remained after filtering the Targetscan database. The thickness of the lines represents the predicted efficacy (cumulative weighted context++ scores) of the miRNA-mRNA interaction. This schematic representation highlights promising miRNA-mRNA interactions that may be potential candidates for HARS associated gene regulation. However, it also demonstrates that several miRNAs and their targeted mRNAs do not show the expected reciprocal expression levels (i.e. down-regulation of miRNA and up-regulation of targeted mRNA. This is also evident for *miR342-3p* which in our study appeared 10-fold down-regulated, but interactions could be found only for down-regulated mRNA and not for up-regulated mRNA (Fig 5, middle panel). These miRNA-mRNA interactions may not be relevant for HARS associated gene regulation or could be false positive predictions. This assumption is corroborated by the fact that cumulative weighted context++ scores were rather low for most of the 211 predicted interactions (data not shown), but for e.g. the down-regulated *miR185-3p* we could find a stronger interaction with up-regulated mRNAs such as *CD177*, *VSIG4*, *FAM101B* (Fig 5, lower panel).

## Discussion

We examined the possible clinical prognostic ability of early radiation-related miRNA gene expression changes in the peripheral blood for the early prediction of late occurring HARS in baboons. We differentiated baboons that developed clinically relevant mild hematological disease (H1-2 degree HARS) as well as baboons that developed more severe hematological disease (H2-3 degree HARS). This discrimination was achieved independently with each of 20 (H1-2°) and 15 (H2-3°) radiation-induced miRNAs examined in the peripheral blood of baboons within the first two days after irradiation.

To our knowledge, we consider our approach to be novel, because our goal was to predict a health outcome (hematologic disease) occurring days and weeks after radiation exposure rather than estimating absorbed dose. We did not seek to supplant biodosimetry, but rather augment it since we assumed that changes in miRNA gene expression would integrate dose and host factors to predict late HARS effects in a baboon model. Our method has the desirable feature of incorporating important modifiers of exposure such as dose rate, homogenous/heterogeneous exposure and partial/total body exposure. Nevertheless, due to the close relationship of the HARS degree with radiation dose (Table 1), the miRNAs at the same time do represent a biodosimetry approach, although the same dose range could cause either a milder (H1-2°) or more aggravated (H2-3°) HARS. This was probably caused since partial body exposure of 30% of the body with e.g. 15 Gy exposure of both arms and the head and a total body exposure with 5 Gy from the biological point of view represent different exposure pattern with a different biological response, but from the physical point of view they are summarized to the same equivalent whole body dose of 5 Gy. Also, inter-individual variance in radiosensitivity cannot be addressed by the exposure height. These aspects are in support our approach to search for bioindicators of the effect. While promising, our results require separate replication by others.

We sought to develop a method to target potentially radiation-exposed individuals into those requiring clinical support. While biodosimetry to estimate radiation dose remains necessary for all who were potentially exposed, it remains essential for clinical resources to focus on identifying individuals who will need hospitalization due to late occurring HARS and individuals developing a more aggravated HARS. These clinical requirements to triage exposed individuals appear to be met with our approach. Furthermore, clinical decision making (hospitalization, therapeutic treatment alternatives) would be enhanced by a reliable diagnosis within the first three days after exposure. Added desirability would be the potential for high-throughput of samples. With the genes identified and the workflow established at our laboratory (automatic RNA isolation and cDNA synthesis combined with the 12 K OpenArray qRT-PCR platform), we would be able to process up to 140 samples per day for HARS prediction.

Eight of the 33 miRNAs we identified code for biological functions such as DNA repair (*miR-145*), migration (*miR-151a-5p*), proliferation/apoptosis (*miR-30d*, *miR-26b-3p*) or are related to radiosensitivity (*miR-20a*, *miR-451*, *miR-744-3p*, S1 Table). Another 11 of the miRNAs are either up-/ or down-regulated after radiation exposure. Fourteen did not reveal annotations or reports of associations with radiation (S1 Table). We wished to understand the miRNA-mRNA relationship so we undertook a bioinformatics interrogation. Our analysis indicated some relationships, but we interpreted the evidence overall as weak. It is more likely that the dysregulated mRNAs identified previously by us in the same baboon model are not under direct control of the miRNAs identified in the present study.

Chromosomal aberrations, such as the dicentric chromosomal fragments, occur after radiation exposure and are a hallmark feature of exposure to ionizing radiation [4,21]. In contrast to other authors and cited work [22–26], we believe that finding expression patterns specific for radiation exposure and subsequent HARS remains challenging. Because of the network nature of the transcriptome, many different influences conspire to produce specific changes in miRNA expression. Nonetheless, the clinical course of HARS is a biological process that presumably must be preceded and controlled by specific miRNA expression changes. Of course, we recognize other factors might also lead to an altered HARS-related miRNA expression. However, it was our goal to search for multiple miRNA candidates that might predict HARS severity irrespective of the exposure type. We used logistic regression to obtain predictive values for H0°, H1-2° or H2-3° HARS severity categories for each of 33 predictor (candidate) miRNAs. These analyses were used to discriminate among unexposed (H0°) and animals who developed mild (H1-2°) or more severe (H2-3°) HARS redundantly. With this approach and the integration of these prediction models we hoped to deal with the network nature of the transcriptome by exploiting redundancies in our prediction models.

Of note, for logistic regression we employed normalized miRNA expression values (as CT-values) and not fold-differences (another blood sample would have been required as a calibrator, which we wanted to avoid). When using normalized miRNA expression values, an individual pre-exposure control is not needed, representing an optimized feature of our methodology. In other words, for prediction of late occurring HARS severity, a blood sample 1–2 days after exposure will be needed, but not a blood sample taken before the exposure occurred.

Our study has limitations. Namely, we performed miRNA expression measurements on qRT-PCR with human genomic sequences, because the baboon transcriptome was not publically available, but the high homology of both genomes (93%) and previous reports by other groups [27–29], supported our approach. Since we used TaqMan chemistry with human primer and probe sequences (high sensitivity and specificity), it is more likely that we lost some of the 667 detectable human miRNAs due to a mismatch with the baboon genome, rather than producing false positives. However, we cannot be completely sure that our candidate miRNAs from the baboon model will be reproducible or equate to humans. Such issues remain to be examined in humans in future experiments.

The 2–4 fold-differences detected for most of our miRNAs are smaller than the 5–10 fold changes we examined in previous analysis using protein coding genes (mRNAs) for HARS prediction. Much more promising was *miR-342-3p*, with a 10-fold difference for HARS 2–3° (versus H0°) and a concordance near 99%. These miRNA species, in combination with some messenger RNAs we already identified using the same baboon model might represent a more robust approach than relying on mRNAs or miRNAs only and it completes the view on transcriptomic (mRNAs) and post-transcriptomic (miRNAs) changes used for prediction and medical management of the HARS. For example, a combinatorial model with mRNA that had up to 30-fold differences among HARS groups (e.g. *WNT3*, *POU2AF1*, *CCR7*, *VSIG4*, *CD177*, *DAGLA*, *ARG2* [15]) in combination with *miR-342-3p* could have improved predictive outcome over using mRNAs or miRNAs alone.

Despite the small sample size of our study, we were able to identify 33 promising miRNAs for HARS prediction. However, future work should consider larger sample sizes and results should be validated in additional species and other clinical settings. Therefore, our candidate miRNAs must be considered as “hypothesis-generating”. Cancer patients undergoing bone marrow transplantation and TBI regimens may be appropriate to consider, with the understanding that their underlying disease will complicate interpretation of gene expression changes. Noteworthy, in preliminary examinations on human samples taken from three leukemia patients 24h after total body irradiation we could already validate 7 out of 9 mRNA species which were identified in our baboon model in previous analysis [15]. Since we used the same analysis strategy for the miRNAs in the current study we are optimistic that some candidate miRNA will be successfully validated in human samples.

In summary, our data support the medical decision making of the HARS even within the first two days after exposure where diagnostic tools for early medical decision are required but so far missing. The miRNA species identified and in particular miR-342-3p add to the previously identified mRNAs and complete the portfolio of identified mRNA and miRNA transcripts for HARS prediction and medical management.

## Supporting Information

S1 Table Summary of the miRNA description and annotation as defined in the miRBase (March 2016) for differentially expressed and validated miRNAs as shown in Table 2 are shown in the left part of the table. Based on a literature search, we also examined the current state of knowledge about the association of these miRNAs with radiation exposure (right part of the table). If only a related microRNA was mentioned in literature, then the name is given in the "effect" column. The search term was the name of the microRNA in the PubMed database plus the term "Radiation". If no results were found the search was repeated with the name of the microRNA only. The titles and abstracts were searched for matches. If no results were found, a PubMed Central® search was done. If mirBase described previous IDs, then the search was repeated for these, too. When using a miRNA acronym combined with the term “Radiation” some important citations were missing, which could be completed thanks to highly appreciated inputs of our unknown reviewers.
(XLS)
